# Exploring the Use of Artificial Intelligence and Wearable Technologies in the Context of Cardiovascular Prevention From Early Detection to Cardiac Recovery: Protocol for a Scoping Review

**DOI:** 10.2196/89602

**Published:** 2026-05-26

**Authors:** Jesús Spósito-Prado, Javier Pereira, Javier Roibal-Pravío

**Affiliations:** 1 Talionis Group Centro de Investigación en Tecnologías de la Información y la Comunicación (CITIC) Universidade da Coruña A Coruña, Galicia Spain

**Keywords:** artificial intelligence, cardiac rehabilitation, cardiovascular prevention, eHealth, primary prevention, remote patient monitoring, secondary prevention, telemedicine, treatment adherence, wearable electronic devices

## Abstract

**Background:**

Cardiac rehabilitation is an evidence-based, multidisciplinary intervention integrating therapeutic exercise, patient education, nutritional counseling, optimized pharmacological management, and psychological support. It reduces cardiovascular mortality and improves functional capacity and quality of life. However, real-world implementation remains suboptimal due to limited accessibility, high dropout rates, and urban-rural inequities. Traditional center-based models rely on in-person supervision, limiting scalability and long-term monitoring beyond structured phases. These limitations are particularly evident during the transition from supervised rehabilitation to long-term self-management, when sustained cardiovascular risk control and maintenance of healthy behaviors are essential. Cardiovascular prevention and cardiac rehabilitation should therefore be understood as interconnected components of a continuum of care. Within this framework, digital health innovations—particularly wearable technologies and artificial intelligence (AI)—offer opportunities to bridge supervised rehabilitation and long-term prevention. Wearables enable continuous remote monitoring of physiological and behavioral parameters, facilitating early detection of adverse events and personalized feedback. AI enhances these capabilities through advanced analysis of clinical and sensor-derived data, supporting risk stratification, prediction of adherence and disease progression, and individualized decision-making across the care pathway.

**Objective:**

This protocol outlines a planned scoping review that will systematically map and characterize the application of AI and wearable technologies across the cardiovascular care continuum—from primary prevention to cardiac rehabilitation—identifying key use cases, technological approaches, research clusters, and knowledge gaps.

**Methods:**

The review will follow the Joanna Briggs Institute methodology for scoping reviews and adhere to PRISMA-ScR (Preferred Reporting Items for Systematic Reviews and Meta-Analyses extension for Scoping Reviews) guidelines. Eligibility criteria are structured according to the Population, Concept, and Context framework. A comprehensive, iteratively developed thesaurus of key terms has been prepared to guide structured searches in PubMed, Web of Science Core Collection, IEEE Xplore, and CINAHL. Two reviewers will independently screen titles, abstracts, and full texts, with disagreements resolved by consensus. A third reviewer will intervene if disagreements persist. Data will be charted using a standardized extraction form and synthesized through narrative synthesis and structured tabulation.

**Results:**

At the time of manuscript submission, the review has not yet commenced. However, a comprehensive thesaurus of key terms has been developed to guide and structure the forthcoming literature search. A preliminary PubMed search retrieved 1255 records; additional database searches are expected to increase this number. No participant recruitment or primary data collection is required. The literature search is planned for April-June 2026, followed by study selection. Results are expected to be available for publication in November 2026.

**Conclusions:**

This review will provide a comprehensive mapping of AI and wearable integration in cardiovascular prevention and rehabilitation, offering an evidence-based foundation for future research and informing digital health strategies aimed at improving long-term cardiovascular outcomes.

**International Registered Report Identifier (IRRID):**

PRR1-10.2196/89602

## Introduction

According to the World Health Organization, cardiac rehabilitation encompasses a set of activities required to ensure that patients with heart disease achieve optimal physical, mental, and social conditions, thereby enabling them to reintegrate into society as normally and independently as possible [1,2].

Today, cardiac rehabilitation programs are typically multidisciplinary in scope, integrating several dimensions of care. Among the most relevant are supervised therapeutic exercise, health education, pharmacotherapy, nutrition, and psychological support. Increasing scientific evidence supports the effectiveness of these programs in reducing cardiovascular mortality, improving functional capacity, and enhancing overall patient well-being [3].

Cardiac rehabilitation is structured into 3 distinct phases, each with specific objectives and targeted to different patient profiles according to their clinical status and individual needs. Phase I, also known as the inpatient or hospital-based phase, takes place during hospitalization after an acute cardiovascular event. Its primary goal is clinical stabilization and preparation of the patient for discharge.

Phase II, the supervised outpatient phase, begins after hospital discharge and focuses on functional recovery. It places particular emphasis on the prescription and monitoring of therapeutic exercise, education on heart-healthy habits, risk factor control, and optimization of medical treatment.

Finally, Phase III, or the maintenance phase, represents a long-term process aimed at consolidating heart-healthy behaviors, maintaining regular physical activity, and ensuring periodic medical follow-up to prevent relapses or new cardiovascular events. In this phase, greater responsibility falls on the patient, who must develop strategies to autonomously adopt and sustain an active lifestyle [4].

The implementation of cardiac rehabilitation programs requires the involvement of a multidisciplinary team of health and exercise professionals. Over time, these programs have expanded to incorporate a broader range of services and to include a variety of specialists, such as cardiologists, exercise medicine physicians, nurses, therapeutic exercise professionals, physiotherapists, psychologists, and nutritionists [5].

Despite their benefits, the implementation of cardiac rehabilitation in clinical practice faces several challenges, including low patient adherence, limited accessibility in certain regions, and difficulties in remote monitoring. Although the benefits of these programs are well documented, dropout rates remain high, undermining their overall effectiveness. Reasons for discontinuation are multiple and complex, including lack of motivation, early perceptions of recovery, insufficient social support, logistical barriers such as distance to rehabilitation centers, and financial constraints [6].

Another critical barrier is the limited geographic availability of these programs, which generates inequities in access to rehabilitation services. In many countries, cardiac rehabilitation centers are concentrated in tertiary hospitals located in large cities, forcing patients from rural areas to travel long distances to receive specialized care. Moreover, the geographic distribution of rehabilitation programs reflects marked global disparities, ranging from 1 center per 4 patients in the Americas to 1 per 283 patients in Southeast Asia and 1 per 529 in Africa [7].

This situation not only hampers attendance at in-person sessions but also negatively affects treatment equity, as patients unable to travel regularly are less likely to benefit from rehabilitation. Furthermore, conventional models rely heavily on face-to-face supervision, limiting opportunities for continuous monitoring of patients’ clinical status outside hospital settings.

In response to these challenges, eHealth solutions—and particularly those leveraging wearable devices and artificial intelligence (AI)—are emerging as innovative tools that can overcome these barriers while improving adherence, effectiveness, and equitable access to cardiac rehabilitation [8]. Beyond rehabilitation, these technologies also play a crucial role in cardiovascular prevention, providing proactive strategies to control risk factors, support healthy lifestyle adoption, and reduce the likelihood of future cardiovascular events. The integration of wearables and AI into both rehabilitation and prevention programs could accelerate the expansion of digital health solutions, which have demonstrated cost-effectiveness and favorable outcomes compared with center-based interventions alone [9,10].

The use of wearable technology enables patients not only to receive personalized guidance, conduct consultations without the need for travel, and access educational resources related to their condition, but also to engage in preventive strategies by continuously monitoring lifestyle-related behaviors and cardiovascular risk factors. Furthermore, wearable devices and biometric sensors are rapidly transforming into core components of both cardiac telerehabilitation and cardiovascular prevention strategies, enabling remote monitoring of physiological variables such as heart rate, blood pressure, heart rate variability, and physical activity levels. This dual application allows for more precise monitoring of patient status, early detection of adverse events, and identification of individuals at high cardiovascular risk [11].

In parallel, AI is providing advanced tools for clinical data processing, risk pattern identification, adherence prediction, and personalized rehabilitation planning. Within the preventive domain, AI-driven models can identify at-risk populations, predict disease trajectories, and generate tailored recommendations to support lifestyle modification and long-term risk factor management. Machine learning algorithms can analyze large volumes of patient data, detect trends over time, and generate automated alerts to support both preventive and rehabilitative decision-making [12].

The combination of wearable devices and AI, therefore, paves the way for the development of intelligent systems that allow the automation of administrative tasks, the personalization of treatment plans, the optimization of workload distribution, and the expansion of both prevention and rehabilitation services [13].

Recent reviews have highlighted the rapid technological evolution of cardiovascular wearables, emphasizing their increasing capacity for multimodal biosensing, real-time data transmission, and integration with advanced analytics pipelines [14,15]. Contemporary devices are no longer limited to basic activity tracking but now enable continuous electrocardiographic monitoring, heart rate variability assessment, and even cuffless blood pressure estimation supported by machine learning algorithms [16]. These advances are reshaping cardiovascular medicine by enabling scalable, longitudinal physiological monitoring in real-world settings and by generating large volumes of high-resolution data suitable for AI-driven interpretation.

At the same time, AI is progressively transforming cardiovascular care at both the individual and population levels. Recent studies have underscored the role of AI in improving cardiovascular population health, optimizing risk stratification, and enhancing early disease detection [17,18]. AI models applied to wearable sensor data have demonstrated promising capabilities for arrhythmia detection, heart failure risk prediction, and cardiovascular event monitoring, although variability in datasets, validation strategies, and outcome definitions remains substantial [19-21].

Beyond rehabilitation contexts, mobile health and wearable technologies are increasingly positioned as central tools in cardiovascular prevention strategies. Evidence indicates that these technologies may facilitate long-term lifestyle modification, medication adherence, and proactive risk management in atherosclerotic cardiovascular disease [22]. Systematic reviews further demonstrate that AI-enabled wearable systems can support remote disease monitoring and decentralized care delivery models, contributing to the expansion of digital cardiology frameworks [23-25]. However, the rapid proliferation of applications across prevention and rehabilitation domains has produced a fragmented evidence landscape characterized by diverse study designs, end points, and technological platforms.

Recent scoping reviews have examined the application of AI in combination with wearable devices for cardiovascular monitoring and disease detection. For example, previous work has explored AI-driven platforms for real-time cardiovascular condition monitoring using wearable sensors, focusing primarily on signal processing pipelines and algorithm deployment for event detection [26]. Other reviews have addressed the broader application of machine learning techniques in cardiovascular disease detection, prediction, and diagnosis, occasionally incorporating wearable-derived data as one of several data sources [27]. While these studies provide valuable insights into the technological capabilities of AI-enabled monitoring systems, their scope has largely centered on diagnostic and real-time monitoring applications. Consequently, the broader integration of AI and wearable technologies across the cardiovascular prevention continuum—including early detection, risk prediction, remote monitoring, and support for cardiac rehabilitation and preventive interventions—remains dispersed across different research domains.

Given this context, we propose conducting a scoping review to comprehensively map existing evidence on the use of AI and wearable devices in cardiac rehabilitation and cardiovascular prevention. AI and wearables are emerging as transformative technologies with the potential to improve treatment personalization, optimize patient adherence, and enhance access to prevention and rehabilitation programs. Considering the heterogeneity of study designs, the rapid evolution of digital health tools, and the emerging yet fragmented nature of the current evidence base, a scoping review is the most appropriate methodology to identify, categorize, and synthesize available knowledge. This approach will facilitate the identification of gaps in the literature and guide the design of future research, providing valuable insights for clinicians, researchers, and policymakers interested in integrating innovative digital solutions into cardiovascular prevention and rehabilitation programs.

## Methods

### Overview

This scoping review follows the methodology developed by the Joanna Briggs Institute (JBI), which is widely recognized as the current reference framework for conducting scoping reviews [28]. The choice of this methodology is based on its suitability for systematically mapping the literature in emerging areas, such as the convergence of cardiovascular prevention, AI, and wearable technology within the fields of health sciences and medical informatics.

The JBI methodology structures the review according to the Population, Concept, and Context (PCC) framework, which supports the exploratory mapping of a broad and heterogeneous body of evidence. In line with the scoping review approach, this methodology does not require risk of bias assessment or meta-analysis, as it aims to provide an overview of the existing literature rather than a critical appraisal or synthesis of effectiveness.

In addition, the development and reporting of this review adhere to the PRISMA-ScR (Preferred Reporting Items for Systematic Reviews and Meta-Analyses extension for Scoping Reviews) guidelines (Multimedia Appendix 1), an extension of the PRISMA (Preferred Reporting Items for Systematic Reviews and Meta-Analyses) statement specifically designed for scoping reviews, ensuring transparency, reproducibility, and comprehensiveness of the final report [29,30].

### Eligibility Criteria

The inclusion criteria for this scoping review have been established using the PCC framework and are designed to ensure alignment with the objectives and scope of the review. The criteria are defined as follows and are summarized in Textbox 1.

**Inclusion and exclusion criteria.**

**Inclusion criteria**

- Studies involving any population in which cardiovascular parameters or outcomes are assessed using artificial intelligence (AI) and/or wearable technologies, regardless of clinical status.
- Studies describing the application, development, evaluation, or implementation of AI and/or wearable technologies in cardiovascular prevention or cardiac rehabilitation.
- Studies conducted in any health care or research setting, including hospital-based, outpatient, community, home-based, or digital/remote environments.
- Studies involving AI methods, wearable technologies, or both, provided they are applied to cardiovascular prevention or cardiac rehabilitation.
- Primary studies (quantitative, qualitative, or mixed methods) and secondary evidence (systematic reviews, meta-analyses, or scoping reviews) with sufficient methodological detail.
- Studies addressing cardiovascular prevention (primary or secondary) and/or cardiac rehabilitation.
- Studies published within the past 5 years.
- Studies published in English or Spanish.
- Published and unpublished studies (eg, preprints, theses, and reports) with accessible full text and sufficient methodological information.

**Exclusion criteria**

- Studies not involving cardiovascular parameters or outcomes.
- Studies focusing exclusively on algorithm development without a clear cardiovascular application, or describing wearable technologies without relevance to cardiovascular prevention or rehabilitation.
- Studies conducted in contexts not related to health care or cardiovascular research.
- Studies not involving AI or wearable technologies.
- Studies lacking sufficient methodological detail to allow characterization or mapping within the review.
- Studies not related to cardiovascular prevention or cardiac rehabilitation.
- Studies published outside the defined time frame.
- Studies published in other languages.
- Studies without an accessible full text or insufficient methodological information.

#### Population

This review will consider studies involving any population in which cardiovascular parameters or outcomes are assessed using AI or wearable technologies, regardless of clinical status.

#### Concept

Eligible sources must explore or describe the application of AI and/or wearable technologies in cardiovascular prevention or rehabilitation. AI refers to computational techniques that support data-driven decision-making (eg, machine learning, deep learning, and predictive algorithms), while wearable technologies include noninvasive sensor-based devices for monitoring physiological parameters. Studies must clearly report on the use, development, evaluation, or implementation of these technologies in the context of cardiovascular care.

#### Context

Any health care or research setting will be considered, including hospital-based, community-based, outpatient, home-based, or digital/remote environments. The review will include both primary prevention settings and rehabilitation contexts, without restriction by geographic location or health care system.

#### Types of Sources

This review will include both primary and secondary sources of evidence. Eligible primary studies may use quantitative, qualitative, or mixed methods designs. Secondary sources, such as systematic reviews, meta-analyses, scoping reviews, and other forms of evidence synthesis, will also be considered when they provide relevant information addressing the review questions. Studies may be published in peer-reviewed journals, conference proceedings, or within the gray literature (eg, theses and government or institutional reports), provided they include sufficient methodological detail to enable appropriate characterization and mapping within the review.

#### Time Frame

Only studies published within the past 5 years will be considered for inclusion, in order to ensure the relevance and currency of evidence in a rapidly evolving technological and clinical landscape.

#### Language

Only studies published in English or Spanish will be considered eligible due to feasibility constraints.

#### Publication Status

Both published and unpublished studies (eg, preprints and theses) will be considered, provided they include sufficient methodological detail to allow assessment of relevance.

#### Operational Definition of Eligible Studies

To ensure consistency during the screening process, additional operational criteria will be applied when determining study eligibility.

Studies will be considered eligible if they report the application, development, or evaluation of AI methods and/or wearable technologies in a context related to cardiovascular prevention or cardiac rehabilitation.

Studies focusing exclusively on algorithm development without a clear cardiovascular application, or studies describing wearable technologies without any relevance to cardiovascular prevention or rehabilitation, will be excluded. Studies involving only wearable technologies without the use of AI will also be considered eligible if they contribute relevant evidence regarding cardiovascular prevention or cardiac rehabilitation. Similarly, studies focusing exclusively on AI methods without the use of wearable technologies will be considered eligible when they address relevant applications in cardiovascular prevention or cardiac rehabilitation, such as cardiovascular risk prediction, clinical decision support, or personalized rehabilitation planning.

Studies addressing both cardiovascular prevention and cardiac rehabilitation will be included. When both domains are present, studies will be categorized according to their primary objective as reported by the authors. If both domains are equally represented, the study will be classified as addressing both prevention and rehabilitation.

For example, studies describing the use of wearable sensors to monitor cardiovascular parameters such as heart rate or blood pressure in ambulatory settings will be considered eligible, as illustrated in recent reviews on wearable technologies for cardiovascular monitoring [13,16]. Studies applying machine learning or other AI techniques to physiological signals obtained from wearable devices to support cardiovascular disease detection or risk prediction will also be considered eligible [21]. In addition, studies applying AI techniques to cardiovascular clinical or population-level data to support cardiovascular risk prediction or population health management, even when wearable devices are not involved, will also be considered eligible [17].

### Information Sources

To ensure a comprehensive and systematic identification of relevant literature, a combination of bibliographic databases and gray literature sources will be consulted. The selected information sources reflect the interdisciplinary nature of the review, encompassing clinical, technological, and public health perspectives. At this stage, no direct contact with study authors is planned.

The following electronic databases will be searched:

- PubMed/MEDLINE for biomedical and clinical literature.
- Web of Science Core Collection for multidisciplinary scientific literature and citation tracking.
- IEEE Xplore for engineering and computer science research related to AI and wearable technologies.
- CINAHL for studies related to nursing, rehabilitation, and allied health fields.

To enhance comprehensiveness and minimize publication bias, this scoping review will include selected sources of gray literature. In particular, searches will be conducted in ProQuest Dissertations & Theses Global to identify relevant academic theses, and in Google Scholar to locate potentially eligible reports, conference proceedings, and nonindexed academic documents. Additionally, recent preprints will be screened through medRxiv and bioRxiv, given their relevance in publishing early biomedical and health-related research, especially in fast-evolving fields such as AI and digital health. Reference lists of included studies and relevant reviews will be screened manually to identify additional sources.

### Search Strategy

The search strategy for this scoping review will follow a 3-step approach that will ensure a comprehensive and systematic identification of both published and unpublished sources of evidence relevant to the topic.

In the first step, an initial limited search will be conducted in at least 2 relevant online databases: PubMed and IEEE Xplore. The purpose of this step is to identify key terms and indexing language commonly used in the literature on AI, wearable technologies, cardiovascular prevention, and cardiac rehabilitation. The titles, abstracts, and index terms of retrieved articles will be analyzed to inform the subsequent full search.

In the second step, a comprehensive search using all identified keywords and index terms will be carried out across all selected databases, which include PubMed, Web of Science Core Collection, IEEE Xplore, and CINAHL. The keywords used for the literature search are detailed in Textbox 2. This search will aim to identify peer-reviewed primary studies and reviews relevant to the research objectives. Boolean operators, truncation, and proximity operators will be used where appropriate, and search strategies will be adapted to the syntax and structure of each database.

**Final thesaurus terms grouped by category.**

**Artificial intelligence (technical terms)**

- active learning; adversarial robustness; AI automation; AI behavioral science; AI data capture; AI data privacy; AI eHealth; AI ethics; AI explainability; AI patient adherence; AI patient monitoring; AI real-time monitoring; AI regulation; AI robotics; AI telemedicine; AI text mining; AI-driven data analytics; AI-enabled medical devices; AI-enabled smartphones; algorithmic bias; algorithmic transparency; cardiac imaging methods; cardiovascular imaging; causal AI; chatbot; ChatGPT; computer vision; convolutional neural networks; deep learning; domain adaptation; domain generalization; explainable artificial intelligence; federated learning; few-shot learning; fuzzy logic; generative adversarial networks; generative artificial intelligence; graph neural networks; image analysis; image interpretation; k-means algorithm; large language models; machine learning; medical imaging; meta-learning; MLOps; natural language processing; pattern recognition; personalized medicine; precision medicine; predictive models; reinforcement learning; self-supervised learning; support vector machines; synthetic data generation; transfer learning; zero-shot learning.

**Wearables (technical terms)**

- bioelectrical impedance sensors; biosensors; Bluetooth Low Energy wearable platforms; edge computing in wearables; electronic skin (e-skin); energy-harvesting wearables; exercise monitoring wearables; flexible/stretchable electronics; implantable wearables; Internet of Medical Things (IoMT); microneedle patches; NFC-enabled wearables; photoplethysmography sensors (PPG); skin-like electronics; smart therapeutic patches/textiles; telemonitoring; vital sign monitoring; wearable biosensors; wearable devices; wearable diagnostics; wearable medical devices; wearable robots; wearable sensors; wearable technology; wearable textiles; wearable ultrasound devices; wireless body area networks (WBAN).

**Cardiovascular prevention (medical terms)**

- aerobic exercise; AI in risk stratification; air pollution; alcohol consumption; anti-inflammatory therapy; arrhythmia; arterial hypertension; arterial motion monitoring; artery calcium score; ASCVD risk score; atherogenesis; atherosclerotic plaque; behavioral change techniques; blood glucose monitoring; blood lipid monitoring; cancer treatments; cardiac function tests; cardiometabolic diseases; cardiovascular digital twin; cardiovascular disease detection; cardiovascular disease diagnosis; cardiovascular disease phenotyping; cardiovascular disease prediction; cardiovascular health literacy; cardiovascular mortality; cardiovascular predictive system; carotid femoral pulse; climate change; coronary disease; coronary syndrome; dietary patterns; digital biomarkers; dyslipidemia; early prevention; early risk factor detection; ECG; environmental pollution; exercise training; exercising; family history of cardiovascular disease; Framingham risk score; geoscience; HbA1c; healthy lifestyle; heart rate zones; heart stroke; imaging biomarkers; imaging techniques; inflammatory disease; insomnia; LDL cholesterol; lifestyle changes; low-density lipoprotein; Mediterranean diet; mindfulness-based interventions; mortality; motivational technologies; multimorbidity; noninvasive measurement techniques; nutritional epidemiology; obesity; ongoing monitoring; oxygen saturation; patient engagement; peripheral vascular disease; personalized training plan; physical activity; physical inactivity; plaque stenosis; prediction models; predictive system; primary cardiovascular prevention; primary prevention; psychosocial factors; real-world data; remote patient monitoring; resistance; respiratory disorders; risk evaluation; risk factors; risk prediction; risk screening; salt consumption; secondary cardiovascular prevention; sedentary behavior; senolytics; sleep apnea; sleep disorders; sleep health; smoking; smoking cessation; social determinants of health; stress levels; stress overload; stroke prevention; training; wearable-based phenotyping; weight management; wellness.

**Cardiac rehabilitation (medical terms)**

- 6-minute walk test (6MWT); accelerometer; adherence rates; ambulatory ECG sensor; anxiety; blood pressure; body composition; body mass index; cardiac rehabilitation program; cardiac rehabilitation screening; cardiac surgery rehabilitation system; cardiological rehabilitation; cardiopulmonary exercise testing (CPET); cardiovascular disease; cardiovascular patients; cardiovascular risk factors; chronotropic incompetence; clinical decision support system; cloud-based rehabilitation dashboard; coaching; congenital heart disease; coronary artery disease; coronary heart disease; depression; diabetes; digital therapeutics platform; ejection fraction; exercise prescription; functional capacity; guided cardiac rehabilitation; heart attack; heart failure; heart rate control; heart rate recovery; heart rate variability; home-based cardiac rehabilitation; hypertension; ischemic heart disease; lifestyle modification; medication adherence; metabolic equivalents (METs); metabolic syndrome; mobile phone; motivational interviewing; myocardial infarction; nutritional counseling; optical flow; patient engagement; patient fatigue; patient portal; peak oxygen uptake (VO₂ peak); pedometer; peripheral vascular disease; phase II; phase III; physical activity counseling; physiological parameters; portable blood pressure monitor; preventive medicine; preserved ejection fraction; psychological intervention; psychosocial assistance; rehabilitation outcome; reduced ejection fraction; risk factor management; robot-assisted therapy; secondary prevention; smart bike; smartphone; social functioning; spirometer; teleconsultation; telehealth platform; telemonitoring; telerehabilitation; ventilatory threshold; weight management.

In the third step, the reference lists of all included full-text articles will be manually screened to identify any additional studies that may not have been captured in the electronic searches. This backward citation tracking will be restricted to the reference lists of studies that are included in the final review after full-text screening.

The search strategy will be developed iteratively, and additional terms may be incorporated as familiarity with the evidence base increases during the review process. All modifications will be transparently documented.

A complete search strategy for the PubMed database will be included in Multimedia Appendix 2 of this protocol. The reviewers recognize the importance of peer reviewing the search strategy, and guidance from the PRESS (Peer Review of Electronic Search Strategies) framework will be followed where feasible. Although no formal peer review of the strategy is planned at this stage, the final search strings will be reviewed internally by the review team, and input from an information specialist will be sought if needed.

### Study Records

#### Data Management

All records identified through database searches and gray literature sources will be imported into a reference management software, such as Mendeley or Zotero. Duplicate entries will be automatically detected and manually reviewed for removal to ensure accuracy. After deduplication, the remaining records will be exported to an online screening platform, such as Covidence or Rayyan, to facilitate title and abstract screening, full-text assessment, and inclusion decision-making.

Throughout the review process, version control and regular backups of datasets will be maintained to safeguard data integrity. Any modifications to the charting form or to inclusion/exclusion decisions will be documented to ensure transparency and reproducibility. The selection process and flow of records will be summarized and reported using a PRISMA-ScR flow diagram.

#### Selection Process

##### Overview

We consulted the official MeSH (Medical Subject Headings) portal to extract all entry terms and synonyms for the 4 concepts of interest: cardiovascular prevention, cardiac rehabilitation, AI, and wearables. We then conducted independent searches in 2 databases, covering the period January 2020-April 2025 and limited to 50 records per concept in each platform (4 concepts × 2 databases × 50 = 400 publications):

In PubMed, the search was conducted using the following parameters:

- Fields: title/abstract.
- Sorting: best match.
- Selection: top 25 most relevant records per concept.
- Screening: titles and abstracts.

In IEEE Xplore, the search was conducted using the following parameters:

- Fields: title, abstract, index terms, author keywords, and IEEE terms.
- Filters: conferences and journals (applied to all 4 concept searches). For the “artificial intelligence” and “wearables” searches only, metadata must include at least one of the following terms: “health,” “medical,” or “clinical.”
- Sorting: relevance.
- Selection: top 25 most relevant records per concept.
- Screening: titles, abstracts, author keywords, and IEEE terms.

To ensure that our IEEE Xplore searches for AI and wearables focus exclusively on health care applications, we applied the metadata filters “health,” “medical,” and “clinical.” After pilot testing, we found that these 3 filters most consistently discriminate clinical applications from general engineering work: “health” captures broad patient well-being interventions; “medical” targets diagnostic or therapeutic systems; and “clinical” identifies studies involving trials, protocols, or real-world care settings.

Next, from the titles, abstracts, and indexed metadata of these 400 references, we manually extracted emerging terms and synonyms. Then, we combined these free-text terms with the MeSH entry terms, performed deduplication (duplicate removal and nomenclature standardization), and finally grouped all surviving keywords into 2 categories: technical terms (AI and wearables) and medical terms (cardiovascular prevention and cardiac rehabilitation).

Furthermore, we refined the original thesaurus by prefixing “AI” to broadly used terms (eg, “AI automation,” “AI telemedicine,” and “AI regulation”) in order to ensure retrieval of publications specifically focused on AI applications. Consequently, this adjustment minimizes unrelated results and enhances the precision of our search strategy without compromising comprehensiveness.

Subsequently, we submitted our initial AI-health thesaurus to the GPT-4 language model (ChatGPT; OpenAI; April 2025 release) for augmentation with emerging trends relevant to cardiac rehabilitation. Specifically, the model’s suggestions—spanning advanced AI methods such as “federated learning,” “self-supervised learning,” “reinforcement learning,” “meta-learning,” “few-shot learning,” “zero-shot learning,” “active learning,” “transfer learning,” “domain adaptation,” “domain generalization,” “multimodal learning,” “graph neural networks,” “causal AI,” “synthetic data generation,” “adversarial robustness,” and “MLOps”—were then independently reviewed and refined by the study authors to ensure methodological rigor and relevance.

In parallel, we submitted our initial wearables thesaurus to the same GPT-4 model for augmentation with emerging wearable technologies relevant to cardiovascular prevention and cardiac rehabilitation. Thereafter, the model’s suggestions—including “edge computing in wearables,” “Internet of Medical Things (IoMT),” “exercise monitoring wearables,” “energy harvesting wearables,” “microneedle patches,” “photoplethysmography sensors (PPG),” “flexible/stretchable electronics,” “bioelectrical impedance sensors,” “NFC-enabled wearables,” and “wearable ultrasound devices”—were likewise reviewed and refined by our team to guarantee both methodological rigor and clinical relevance.

In addition to these AI and wearables efforts, we applied a parallel and equally rigorous process to develop the cardiac prevention thesaurus. To further enrich this resource, this thesaurus was independently submitted to GPT-4 to identify high-impact emerging terms specifically related to prevention strategies. The model suggested the inclusion of concepts such as: “AI in risk stratification,” “ASCVD risk score,” “behavioral change techniques,” “blood glucose monitoring,” “cardiovascular digital twin,” “cardiovascular disease phenotyping,” “cardiovascular health literacy,” “dietary patterns,” “digital biomarkers,” “family history of cardiovascular disease,” “Framingham risk score,” “HbA1c,” “mindfulness-based interventions,” “mobile health (mHealth),” “motivational technologies,” “multimorbidity,” “real-world data,” “sedentary behavior,” “sleep apnea,” “social determinants of health,” and “wearable-based phenotyping.” These terms were reviewed and refined by the authors to ensure methodological rigor and domain relevance.

In the next step, from the initial pool, duplicates and near-synonyms were collapsed into single preferred terms. Overly generic entries (eg, “application,” “sensor,” and “messaging system”) have been retained but will be explicitly paired with the modifiers cardiac or heart in the final search string to prevent off-topic retrieval. Detailed intervention descriptors were set aside for manual screening rather than included in the primary search string.

Moreover, recognizing that a gap existed in explicit digitalization concepts, we queried GPT-4 and received the following specific suggestions: “telehealth platform”; “digital therapeutics platform”; “cloud-based rehabilitation dashboard”; “patient portal”; “telemonitoring gateway”; “clinical decision support system.” These ChatGPT-proposed terms were then independently reviewed and refined to ensure methodological rigor and domain relevance. Finally, all remaining terms were organized alphabetically into a single master list.

Lastly, both the prevention and cardiac rehabilitation thesauri were cross-reviewed to identify and remove duplicative or overlapping entries.

### Use of GPT-4 to Refine Search Terminology

#### Overview

To improve the sensitivity of the search strategy, we used a large language model (LLM) as an auxiliary tool to suggest additional candidate search terms and synonyms. Specifically, we used GPT-4 (OpenAI) in April 2025. The LLM was used only to propose terms; the final selection was made by the review team according to predefined rules (subsection “ Decision rules for accepting or rejecting LLM suggestions” below). No automated inclusion of terms was performed.

#### Prompt (Verbatim)

The exact prompt used to expand the “cardiac rehabilitation” term set was:

“You are an expert in cardiac rehabilitation and cardiovascular prevention. The following list of concepts was generated after reviewing the 100 most relevant articles on cardiac rehabilitation indexed in PubMed and IEEE Xplore between 2020 and 2025.

This list will be used to construct a search strategy that will later be combined with terms related to AI and wearable technologies in order to identify studies located at the intersection of these fields.

Please review the list and indicate whether any relevant concepts related to cardiac rehabilitation may be missing. If so, suggest additional terms that could improve the comprehensiveness of the search strategy.”

We applied the same prompt structure (identical wording with the topic substituted) to expand the term sets for “cardiovascular prevention,” “wearables,” and “artificial intelligence.”

#### Decision Rules for Accepting or Rejecting LLM Suggestions

All LLM-proposed terms were independently screened by 2 reviewers (JS-P and JP) and incorporated into the search strategy only if they met all of the following criteria:

- Conceptual relevance: the term clearly represented a concept within the target domain (cardiac rehabilitation, cardiovascular prevention, wearables, or AI) and could plausibly retrieve eligible studies.
- Nonredundancy: exact duplicates and purely stylistic variants were removed. When several near-synonyms existed, we retained the most standard label and kept alternative spellings only if they were commonly used in indexing or in titles/abstracts.
- Terminological validity: the term reflected established usage in the field, verified against at least one of the following: MeSH/Emtree (when applicable), standard clinical terminology, or occurrence in the seed set of highly relevant articles.
- Scope alignment: overly broad terms likely to generate substantial irrelevant retrieval (eg, generic “rehabilitation” without cardiac context) were excluded unless paired with cardiac qualifiers in the final query.

Disagreements were resolved by discussion and, when needed, adjudication by a third reviewer (JR-P).

#### Model/Version and Date

We used GPT-4 in April 2025. The ChatGPT interface did not provide a persistent snapshot identifier; therefore, we report the verbatim prompts and the predefined rules used to accept/reject suggestions.

### Thesaurus Validation Phase

To ensure terminological coherence and minimize potential bias, the thesaurus was independently validated by the authors through blinded review of a 20% random sample (n=40). Although the reviewers were involved in thesaurus development, each conducted extraction and classification independently. Discrepancies were resolved through consensus, followed by a final audit of the complete list to refine terminology, eliminate residual duplicates, and confirm the classification into technical and medical term categories.

### Data Collection Process

Two independent reviewers (JS-P and JP) will screen titles and abstracts using the selected screening platform, following the predefined eligibility criteria. Any discrepancies will be resolved through discussion and consensus; a third reviewer (JR-P) will intervene if disagreements persist. Full-text articles selected for inclusion will be uploaded and managed collaboratively within the same platform, where inclusion and exclusion decisions will be documented.

Data extraction will be carried out using a predefined charting form developed in Microsoft Excel or Google Sheets. The form will be piloted on a small sample of studies to ensure clarity and consistency. All data entries will be cross-checked by a second reviewer (JP) to confirm accuracy and minimize transcription errors.

### Data Items

In accordance with the methodological approach of scoping reviews, items 13-17 of the PRISMA-P (Preferred Reporting Items for Systematic Review and Meta-Analysis Protocols) checklist related to quantitative synthesis and critical appraisal are not applicable to this protocol. Specifically, this review will not involve prioritization of outcomes, assessment of risk of bias in individual studies, quantitative data synthesis, evaluation of meta-biases, or appraisal of the overall certainty of evidence. These elements are not consistent with the objectives of a scoping review, which aim to map and characterize the existing body of literature rather than to estimate intervention effects or determine the strength of evidence, as described in the JBI methodology and PRISMA-ScR reporting guidelines.

Nevertheless, relevant descriptive and analytical information will be extracted from each included study to enable systematic mapping of the evidence. Extracted variables will include the year of publication, country of origin, study design, target population, cardiovascular condition or prevention context addressed, type of digital technology used, and key findings reported by the authors.

In addition, specific information describing the functional role of AI and wearable technologies will be collected. For each study, reviewers will record the technological purpose of the system, the cardiovascular domain addressed (eg, prevention, risk assessment, monitoring, or rehabilitation), the clinical or preventive objective of the technology, the type of physiological or clinical data used, and the practical application described in the study. Collectively, these variables will allow the review to characterize how digital technologies are applied within cardiovascular prevention and cardiac rehabilitation and will support the subsequent identification of technological use patterns across the literature.

### Data Synthesis

Quantitative synthesis (meta-analysis) will not be conducted in this review. The purpose of this scoping review is to map and categorize the available evidence rather than to estimate pooled effect sizes or evaluate intervention effectiveness. Consequently, statistical pooling, subgroup analyses, and sensitivity analyses are not planned. Instead, the evidence will be synthesized using descriptive and thematic approaches consistent with the methodological framework of scoping reviews.

Data from the included studies will be extracted using a structured data charting form developed specifically for this review. The charting form will be piloted on a small sample of studies to ensure clarity, consistency, and completeness of data extraction. If necessary, the form will be refined during the review process. Data extraction will be performed by one reviewer (JS-P) and verified by a second reviewer (JP) to ensure accuracy and methodological consistency.

For each included study, the following information will be charted. Additional variables may be incorporated if deemed relevant during the iterative review process:

- Bibliographic information (authors, year of publication, and country)
- Study design and methodological approach
- Characteristics of the study population
- Cardiovascular condition or prevention context addressed
- Type of digital technology used (eg, wearable devices, AI algorithms, or integrated systems)
- Type of AI technique used (when applicable)
- Physiological parameters or outcomes monitored
- Main objectives of the study
- Key findings and reported applications
- Limitations reported by the authors

Following data extraction, the evidence will be synthesized in several stages. First, a descriptive analysis will summarize the distribution of included studies according to publication year, geographic region, study design, population characteristics, and technological approaches.

Second, studies will be categorized according to their primary technological and clinical application domains, interpreted as practical use cases describing how AI and wearable technologies are implemented in cardiovascular prevention and cardiac rehabilitation. These domains may include, but are not limited to:

- Cardiovascular risk prediction and prognostic modeling
- Cardiovascular disease detection and diagnosis using AI
- Remote monitoring of physiological signals using wearable devices
- Signal processing and digital biomarker extraction from wearable data
- Digital cardiac rehabilitation and intervention delivery
- AI-supported clinical decision-making systems
- Disease-specific applications (eg, heart failure and atrial fibrillation)
- Behavioral and lifestyle interventions for cardiovascular prevention

The identification of these technological and clinical application domains was informed by an exploratory analysis of the literature retrieved through a preliminary search conducted in PubMed using the search strategy described in Multimedia Appendix 2.

Titles and available metadata from the retrieved records were examined to identify recurrent patterns in the reported applications of AI and wearable technologies within cardiovascular prevention and cardiac rehabilitation. This process allowed the identification of frequently occurring functional uses (eg, prediction, detection, and monitoring), types of data and signals (eg, electrocardiographic and physiological parameters), and clinical contexts (eg, heart failure and atrial fibrillation).

Third, an inductive thematic analysis will be conducted to identify recurring research themes across the included studies. Based on similarities in technological purpose, implementation context, and clinical application, studies will be grouped into conceptual research clusters representing the principal areas of technological development within the field.

Finally, knowledge gaps will be identified by examining underrepresented technological approaches, clinical contexts, study designs, and patient populations within the mapped evidence base.

The results will be presented through descriptive summaries, thematic categorizations, and evidence mapping, including summary tables, thematic diagrams, and narrative synthesis describing the identified technological use cases and research clusters.

### Ethics, Transparency, and Responsible Use of AI

#### Overview

This study constitutes a scoping review of publicly available literature and does not involve human participants, identifiable personal data, or access to confidential information. Therefore, in accordance with institutional and international research ethics guidelines, formal ethics committee approval is not required.

#### Responsible Use of AI

An LLM (GPT-4) was used exclusively as an auxiliary tool to support the refinement of search terminology during the development of the search strategy. The model was used solely to suggest potential additional keywords and synonyms. No automated inclusion of terms was performed. All AI-generated suggestions were independently screened by 2 reviewers (JS-P and JP) according to predefined decision rules, and final decisions were made exclusively by the research team.

AI tools may be used in a limited supportive capacity (eg, for terminology refinement during search strategy development). However, AI systems will not be used to autonomously conduct study screening, eligibility assessment, data extraction, critical appraisal, data synthesis, or interpretation of findings. All methodological decisions will remain under full human oversight and responsibility.

#### Data Handling and Management

The review will use bibliographic metadata and full-text articles obtained through institutional subscriptions and open-access sources. No personal or sensitive data will be processed. All data will be handled in accordance with institutional data protection policies and applicable regulations.

#### Gray Literature and Copyright Compliance

Gray literature sources will be identified using predefined and transparent search procedures. Screening and eligibility assessment will be conducted manually by the review team. No automated scraping of restricted databases or proprietary platforms will be performed. Full-text materials will be accessed exclusively through legally authorized channels, including institutional subscriptions and open-access repositories. AI tools will not be used to reproduce, redistribute, or store copyrighted materials beyond standard academic use.

#### Use of Generative AI in Manuscript Preparation

Generative AI (GPT-4) was used in a limited and clearly defined capacity during the preparation of this manuscript. Specifically, the tool was used to support language refinement and improve clarity of expression in selected sections of the text, as well as to assist in drafting and organizing the initial structure of the thesaurus developed to construct the search strategy.

The AI system was not used to generate scientific conclusions, interpret findings, conduct data analysis, determine eligibility criteria, or make methodological decisions. All conceptual development, methodological design, validation of terminology, and final content were developed, critically reviewed, and approved exclusively by the authors.

All AI-assisted outputs were carefully examined and revised by the research team prior to inclusion. The authors take full responsibility for the accuracy, integrity, and originality of the manuscript.

## Results

**Figure 1:**
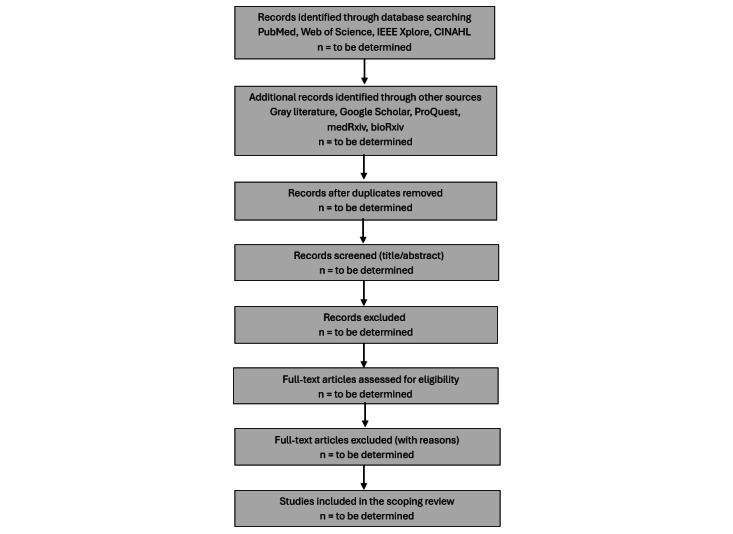
Planned PRISMA-ScR (Preferred Reporting Items for Systematic Reviews and Meta-Analyses extension for Scoping Reviews) flow diagram for study selection. This preliminary diagram illustrates the planned study selection process. Placeholder values are presented, and final numbers will be reported in the completed scoping review in accordance with PRISMA-ScR guidelines.

The literature search is scheduled to begin in April 2026 and is expected to be completed by June 2026. Study selection, data extraction, and thematic analysis will be conducted between July 2026 and October 2026. At the time of manuscript submission, data collection has not yet started. Results are expected to be available for publication in November 2026. The study selection process will be conducted following PRISMA-ScR guidelines. A preliminary flow diagram illustrating the planned selection process is presented in Figure 1.

The results of the scoping review will be presented using both narrative descriptions and structured tables to ensure comprehensive and transparent mapping of the extracted data. Initially, a descriptive summary will outline the characteristics of included studies, contextualizing them according to the review objectives and the PCC framework.

Data will be charted in tables that categorize studies by type of AI or wearable technology applied, target population (eg, general population and cardiac patients), preventive or rehabilitative purpose, health care setting, and methodological design. This tabular synthesis will facilitate the identification of research clusters, emerging areas of interest, and potential knowledge gaps across domains.

Where appropriate, results will also be presented visually using figures or conceptual maps to illustrate the distribution of research over time, thematic intersections between AI, wearables, and cardiovascular care, or geographic patterns. The structure and format of tables and visual summaries may be refined during the review process as familiarity with the evidence base increases.

This approach aims to provide a transparent and structured overview of the existing evidence while supporting future research planning and policy development in digital cardiovascular health.

## Discussion

This scoping review will systematically map the existing literature on the application of AI and wearable technologies in cardiovascular prevention and cardiac rehabilitation. Given the rapid expansion and multidisciplinary nature of this field, a comprehensive synthesis is critical to identify current trends, research clusters, and knowledge gaps that can inform clinical practice, policy, and future investigations.

The review will help identify research clusters, underexplored areas, and methodological gaps that may inform future systematic reviews or primary studies. It will also provide a foundation for designing digital health interventions tailored to the needs of people with cardiac conditions and at-risk populations, especially in preventive and rehabilitative contexts.

By following the JBI methodology and adhering to the PRISMA-ScR guidelines, this review is designed to ensure methodological rigor, transparency, and reproducibility. The integration of a robust AI-augmented thesaurus and a multidatabase search strategy, including gray literature sources, is expected to enhance the comprehensiveness of evidence retrieval. The use of structured tables and visual representations will facilitate a clear understanding of how AI and wearable technologies are being implemented across different populations and health care settings.

One of the anticipated strengths of this review lies in its interdisciplinary scope, capturing evidence from both health sciences and engineering perspectives. However, certain limitations are expected. The review will be restricted to studies published in English and Spanish, which may exclude relevant work in other languages. Additionally, by focusing on literature from the past 5 years, there is a possibility of omitting foundational studies that predate recent technological advances but remain contextually important.

Despite these limitations, the outcomes of this review will provide a valuable reference point for stakeholders involved in the design, implementation, and evaluation of digital health interventions for cardiovascular care. It will also help guide future systematic reviews and empirical studies by identifying methodological and thematic gaps in the current evidence base.

The findings of this scoping review will be disseminated through a peer-reviewed publication in a high-impact journal focused on digital health, cardiovascular prevention, or biomedical informatics. In addition, results will be presented at national and international conferences related to AI in health care and cardiovascular research. Summaries may also be made available through institutional repositories and open-access platforms to support knowledge translation among researchers, clinicians, and policymakers.

Ultimately, the findings of this scoping review may support the design of innovative digital health strategies and contribute to the formulation of policy recommendations in the domain of cardiovascular prevention and rehabilitation.

## Appendix

Multimedia Appendix 1 PRISMA-ScR checklist used to guide the reporting of this scoping review protocol.

Multimedia Appendix 2 Full PubMed search strategy, including all search blocks and Boolean combinations used for study identification.

## Abbreviations

AI: artificial intelligence
JBI: Joanna Briggs Institute
LLM: large language model
MeSH: Medical Subject Headings
PCC: Population, Concept, and Context
PRESS: Peer Review of Electronic Search Strategies
PRISMA: Preferred Reporting Items for Systematic Reviews and Meta-Analyses
PRISMA-P: Preferred Reporting Items for Systematic Review and Meta-Analysis Protocols
PRISMA-ScR: Preferred Reporting Items for Systematic Reviews and Meta-Analyses extension for Scoping Reviews

## Data Availability

This is a protocol for a scoping review. No datasets have yet been generated or analyzed. The review will rely exclusively on data from published studies cited in the manuscript.

## References

[1] Brown RA (1964). Rehabilitation of patients with cardiovascular diseases. Report of a WHO expert committee. World Health Organ Tech Rep Ser.

[2] NA (1993). Rehabilitation after cardiovascular diseases, with special emphasis on developing countries. Report of a WHO expert committee. World Health Organ Tech Rep Ser.

[3] Dalal HM, Doherty P, Taylor RS (2015). Cardiac rehabilitation. BMJ.

[4] Cowie A, Buckley J, Doherty P, Furze G, Hayward J, Hinton S, et al (2019). Standards and core components for cardiovascular disease prevention and rehabilitation. Heart.

[5] Brown TM, Pack QR, Aberegg E, Brewer LC, Ford YR, Forman DE, et al (2024). Core components of cardiac rehabilitation programs: 2024 update: a scientific statement from the American Heart Association and the American Association of Cardiovascular and Pulmonary Rehabilitation. Circulation.

[6] Fukumoto Y (2026). Barriers to participation in cardiac rehabilitation. Eur J Prev Cardiol.

[7] Taylor RS, Fredericks S, Jones I, Neubeck L, Sanders J, De Stoutz N, et al (2023). Global perspectives on heart disease rehabilitation and secondary prevention: a scientific statement from the Association of Cardiovascular Nursing and Allied Professions, European Association of Preventive Cardiology, and International Council of Cardiovascular Prevention and Rehabilitation. Eur Heart J.

[8] Golbus JR, Lopez-Jimenez F, Barac A, Cornwell WK, Dunn P, Forman DE, et al (2023). Digital technologies in cardiac rehabilitation: a science advisory from the American Heart Association. Circulation.

[9] Brouwers RWM, van der Poort EKJ, Kemps HMC, van den Akker-van Marle ME, Kraal JJ (2021). Cost-effectiveness of cardiac telerehabilitation with relapse prevention for the treatment of patients with coronary artery disease in the Netherlands. JAMA Netw Open.

[10] Nkonde-Price C, Reynolds K, Najem M, Yang S, Batiste C, Cotter T, et al (2022). Comparison of home-based vs center-based cardiac rehabilitation in hospitalization, medication adherence, and risk factor control among patients with cardiovascular disease. JAMA Netw Open.

[11] Antoniou V, Davos CH, Kapreli E, Batalik L, Panagiotakos DB, Pepera G (2022). Effectiveness of home-based cardiac rehabilitation, using wearable sensors, as a multicomponent, cutting-edge intervention: a systematic review and meta-analysis. J Clin Med.

[12] Raita Y, Goto T, Faridi MK, Brown DFM, Camargo CA, Hasegawa K (2023). Artificial intelligence in cardiology: applications and obstacles. Comput Methods Programs Biomed.

[13] Williams GJ, Al-Baraikan A, Rademakers FE, Ciravegna F, van de Vosse FN, Lawrie A, et al (2023). Wearable technology and the cardiovascular system: the future of patient assessment. Lancet Digit Health.

[14] Hughes A, Shandhi MMH, Master H, Dunn J, Brittain E (2023). Wearable devices in cardiovascular medicine. Circ Res.

[15] Iqbal SMA, Leavitt MA, Mahgoub I, Asghar W (2024). Advances in cardiovascular wearable devices. Biosensors (Basel).

[16] Min S, An J, Lee JH, Kim JH, Joe DJ, Eom SH, et al (2025). Wearable blood pressure sensors for cardiovascular monitoring and machine learning algorithms for blood pressure estimation. Nat Rev Cardiol.

[17] Meder B, Asselbergs FW, Ashley E (2025). Artificial intelligence to improve cardiovascular population health. Eur Heart J.

[18] Elias P, Jain SS, Poterucha T, Randazzo M, Lopez Jimenez F, Khera R, et al (2024). Artificial intelligence for cardiovascular care-part 1: advances: JACC review topic of the week. J Am Coll Cardiol.

[19] Elvas LB, Almeida A, Ferreira JC (2025). The role of AI in cardiovascular event monitoring and early detection: scoping literature review. JMIR Med Inform.

[20] Lee S, Chu Y, Ryu J, Park YJ, Yang S, Koh SB (2022). Artificial intelligence for detection of cardiovascular-related diseases from wearable devices: a systematic review and meta-analysis. Yonsei Med J.

[21] Dhingra Lovedeep S, Aminorroaya Arya, Pedroso Aline F, Khunte Akshay, Sangha Veer, McIntyre Daniel, Chow Clara K, Asselbergs Folkert W, Brant Luisa C C, Barreto Sandhi M, Ribeiro Antonio Luiz P, Krumholz Harlan M, Oikonomou Evangelos K, Khera Rohan (2025). Artificial Intelligence-Enabled Prediction of Heart Failure Risk From Single-Lead Electrocardiograms. JAMA Cardiol.

[22] Alipour P, El-Aghil M, Foo A, Azizi Z (2025). Leveraging mobile health and wearable technologies for the prevention and management of atherosclerotic cardiovascular disease. Curr Atheroscler Rep.

[23] Spethmann S, Hindricks G, Koehler K, Stoerk S, Angermann CE, Böhm M, et al (2024). Telemonitoring for chronic heart failure: narrative review of the 20-year journey from concept to standard care in Germany. J Med Internet Res.

[24] Wang R, Veera SCM, Asan O, Liao T (2024). A systematic review on the use of consumer-based ECG wearables on cardiac health monitoring. IEEE J Biomed Health Inform.

[25] Triantafyllidis A, Kondylakis H, Katehakis D, Kouroubali A, Koumakis L, Marias K, et al (2022). Deep learning in mHealth for cardiovascular disease, diabetes, and cancer: systematic review. JMIR Mhealth Uhealth.

[26] Abedi A, Verma A, Jain D, Kaetheeswaran J, Chui C, Lankarany M, et al (2025). AI-driven real-time monitoring of cardiovascular conditions with wearable devices: scoping review. JMIR Mhealth Uhealth.

[27] Huang JD, Wang J, Ramsey E, Leavey G, Chico TJA, Condell J (2022). Applying artificial intelligence to wearable sensor data to diagnose and predict cardiovascular disease: a review. Sensors (Basel).

[28] Peters MDj, Godfrey C, McInerney P, Munn Z, Tricco AC, Khalil H (2024). Scoping reviews. JBI Manual for Evidence Synthesis.

[29] Moher D, Shamseer L, Clarke M, Ghersi D, Liberati A, Petticrew M, et al (2015). Preferred reporting items for systematic review and meta-analysis protocols (PRISMA-P) 2015 statement. Syst Rev.

[30] Shamseer L, Moher D, Clarke M, Ghersi D, Liberati A, Petticrew M, et al (2015). Preferred reporting items for systematic review and meta-analysis protocols (PRISMA-P) 2015: elaboration and explanation. BMJ.

