# Supplementary material for: Exploring the Use of Artificial Intelligence and Wearable Technologies in the Context of Cardiovascular Prevention From Early Detection to Cardiac Recovery: Protocol for a Scoping Review
Source: JMIR Res Protoc. 2026 May 26;15:e89602. doi: 10.2196/89602 (PMC13250499; doi:10.2196/89602)
Supplement: Multimedia Appendix 2 [file resprot_v15i1e89602_app2.pdf]

APPENDIX 2. PUBMED SEARCH STRATEGY

This appendix presents the complete PubMed search strategy derived from the structured thesaurus. All terms were searched in the Title/Abstract field. The strategy was designed for reproducibility and transparency and will be adapted to other databases (Web of Science, IEEE Xplore, CINAHL).

The search combines four blocks: Artificial Intelligence (Block A) and Wearables (Block B), intersected with either Cardiovascular Prevention (Block C) or Cardiac Rehabilitation (Block D).

BLOCK A – ARTIFICIAL INTELLIGENCE
("Artificial Intelligence"[Title/Abstract] OR "Active Learning"[Title/Abstract] OR "Adversarial Robustness"[Title/Abstract] OR "AI Automation"[Title/Abstract] OR "AI Behavioral Science"[Title/Abstract] OR "AI Data Capture"[Title/Abstract] OR "AI Data Privacy"[Title/Abstract] OR "AI Ehealth"[Title/Abstract] OR "AI Ethics"[Title/Abstract] OR "AI Explainability"[Title/Abstract] OR "AI Patient Adherence"[Title/Abstract] OR "AI Patient Monitoring"[Title/Abstract] OR "AI Real-Time Monitoring"[Title/Abstract] OR "AI Regulation"[Title/Abstract] OR "AI Robotics"[Title/Abstract] OR "AI Telemedicine"[Title/Abstract] OR "AI Text Mining"[Title/Abstract] OR "AI-driven Data Analytics"[Title/Abstract] OR "AI-enabled Medical Devices"[Title/Abstract] OR "AI-enabled Smart Phones"[Title/Abstract] OR "Algorithmic Bias"[Title/Abstract] OR "Algorithmic Transparency"[Title/Abstract] OR "Cardiac Imaging Methods"[Title/Abstract] OR "Cardiovascular Imaging"[Title/Abstract] OR "Causal AI"[Title/Abstract] OR "Chatbot"[Title/Abstract] OR "Chatgpt"[Title/Abstract] OR "Computer Vision"[Title/Abstract] OR "Convolutional Neural Networks"[Title/Abstract] OR "Deep Learning"[Title/Abstract] OR "Domain Adaptation"[Title/Abstract] OR "Domain Generalization"[Title/Abstract] OR "Explainable Artificial Intelligence"[Title/Abstract] OR "Federated Learning"[Title/Abstract] OR "Few-shot Learning"[Title/Abstract] OR "Fuzzy Logic"[Title/Abstract] OR "Generative Adversarial Networks"[Title/Abstract] OR "Generative Artificial Intelligence"[Title/Abstract] OR "Graph Neural Networks"[Title/Abstract] OR "Image Analysis"[Title/Abstract] OR "Image Interpretation"[Title/Abstract] OR "K-means Algorithm"[Title/Abstract] OR "Large Language Models"[Title/Abstract] OR "Machine Learning"[Title/Abstract] OR "Medical Imaging"[Title/Abstract] OR "Meta-learning"[Title/Abstract] OR "Mlops"[Title/Abstract] OR "Natural Language Processing"[Title/Abstract] OR "Pattern Recognition"[Title/Abstract] OR "Personalized Medicine"[Title/Abstract] OR "Precision Medicine"[Title/Abstract] OR "Predictive Models"[Title/Abstract] OR "Reinforcement Learning"[Title/Abstract] OR "Self-supervised Learning"[Title/Abstract] OR "Support Vector Machines"[Title/Abstract] OR "Synthetic Data Generation"[Title/Abstract] OR "Transfer Learning"[Title/Abstract] OR "Zero-shot Learning"[Title/Abstract])

BLOCK B – WEARABLES
("Wearables"[Title/Abstract] OR "Bioelectrical Impedance Sensors"[Title/Abstract] OR "Biosensors"[Title/Abstract] OR "Bluetooth Low Energy Wearable Platforms"[Title/Abstract] OR "Edge Computing In Wearables"[Title/Abstract] OR "Electronic Skin"[Title/Abstract] OR "Energy Harvesting Wearables"[Title/Abstract] OR "Exercise Monitoring Wearables"[Title/Abstract] OR "Flexible/stretchable Electronics"[Title/Abstract] OR "Implantable Wearables"[Title/Abstract] OR "Internet Of Medical Things"[Title/Abstract] OR "Microneedle Patches"[Title/Abstract] OR "Nfc-enabled Wearables"[Title/Abstract] OR "Photoplethysmography Sensors"[Title/Abstract] OR "Skin-like Electronics"[Title/Abstract] OR "Smart Therapeutic Patches/textiles"[Title/Abstract] OR "Telemonitoring"[Title/Abstract] OR "Vital Sign Monitoring"[Title/Abstract] OR "Wearable Bionsensors"[Title/Abstract] OR "Wearable Devices"[Title/Abstract] OR "Wearable Diagnostics"[Title/Abstract] OR "Wearable Medical Devices"[Title/Abstract] OR "Wearable Robots"[Title/Abstract] OR "Wearable Sensors"[Title/Abstract] OR "Wearable Technology"[Title/Abstract] OR "Wearable Textiles"[Title/Abstract] OR "Wearable Ultrasound Devices"[Title/Abstract] OR "Wireless Body Area Networks"[Title/Abstract])

BLOCK C – CARDIOVASCULAR PREVENTION

("Cardiovascular Prevention"[Title/Abstract] OR "Aerobic Exercise"[Title/Abstract] OR "Ai In Risk Stratification"[Title/Abstract] OR "Air Pollution"[Title/Abstract] OR "Alcohol Consumption"[Title/Abstract] OR "Anti-inflammatory Therapy"[Title/Abstract] OR "Arrythmia"[Title/Abstract] OR "Arterial Hypertension"[Title/Abstract] OR "Arterial Motion Monitoring"[Title/Abstract] OR "Artery Calcium Score"[Title/Abstract] OR "Ascvd Risk Score"[Title/Abstract] OR "Atherogenesis"[Title/Abstract] OR "Atherosclerotic Plaque"[Title/Abstract] OR "Behavioral Change Techniques"[Title/Abstract] OR "Blood Glucose Monitoring"[Title/Abstract] OR "Blood Lipid Monitoring"[Title/Abstract] OR "Cancer Treatments"[Title/Abstract] OR "Cardiac Function Tests"[Title/Abstract] OR "Cardiometabolic Diseases"[Title/Abstract] OR "Cardiovascular Digital Twin"[Title/Abstract] OR "Cardiovascular Disease Detection"[Title/Abstract] OR "Cardiovascular Disease Diagnosis"[Title/Abstract] OR "Cardiovascular Disease Phenotyping"[Title/Abstract] OR "Cardiovascular Disease Prediction"[Title/Abstract] OR "Cardiovascular Health Literacy"[Title/Abstract] OR "Cardiovascular Mortality"[Title/Abstract] OR "Cardiovascular Predictive System"[Title/Abstract] OR "Carotid Femoral Pulse"[Title/Abstract] OR "Climate Change"[Title/Abstract] OR "Coronary Disease"[Title/Abstract] OR "Coronary Syndrome"[Title/Abstract] OR "Dietary Patterns"[Title/Abstract] OR "Digital Biomarkers"[Title/Abstract] OR "Dyslipidemia"[Title/Abstract] OR "Early Prevention"[Title/Abstract] OR "Early Risk Factor Detection"[Title/Abstract] OR "Ecg"[Title/Abstract] OR "Environmental Pollution"[Title/Abstract] OR "Exercise Training"[Title/Abstract] OR "Exercising"[Title/Abstract] OR "Family History Of Cardiovascular Disease"[Title/Abstract] OR "Framingham Risk Score"[Title/Abstract] OR "Geoscience"[Title/Abstract] OR "Hba1c"[Title/Abstract] OR "Health Lifestyle"[Title/Abstract] OR "Heart Rate Zones"[Title/Abstract] OR "Heart Stroke"[Title/Abstract] OR "Imaging Biomarkers"[Title/Abstract] OR "Imaging Techniques"[Title/Abstract] OR "Inflammatory Disease"[Title/Abstract] OR "Insomnia"[Title/Abstract] OR "Ldl-cholesterol"[Title/Abstract] OR "Lifestyle Changes"[Title/Abstract] OR "Low-density Lipoprotein"[Title/Abstract] OR "Mediterranean Diet"[Title/Abstract] OR "Mindfullness-based Interventions"[Title/Abstract] OR "Mortality"[Title/Abstract] OR "Motivational Technologies"[Title/Abstract] OR "Multimorbidity"[Title/Abstract] OR "Non-invasive Measurement Techniques"[Title/Abstract] OR "Nutritional Epidemiology"[Title/Abstract] OR "Obesity"[Title/Abstract] OR "Ongoing Monitoring"[Title/Abstract] OR "Oxygen Saturation"[Title/Abstract] OR "Patient Engagement"[Title/Abstract] OR "Peripheral Vascular Disease"[Title/Abstract] OR "Personalized Training Plan"[Title/Abstract] OR "Physical Activity"[Title/Abstract] OR "Physical Inactivity"[Title/Abstract] OR "Plaque Stenosis"[Title/Abstract] OR "Prediction Models"[Title/Abstract] OR "Predictive System"[Title/Abstract] OR "Primary Cardiovascular Prevention"[Title/Abstract] OR "Primary Prevention"[Title/Abstract] OR "Psychosocial Factors"[Title/Abstract] OR "Real-world Data"[Title/Abstract] OR "Remote Patient Monitoring"[Title/Abstract] OR "Resistance"[Title/Abstract] OR "Respiratory Disorders"[Title/Abstract] OR "Risk Evaluation"[Title/Abstract] OR "Risk Factors"[Title/Abstract] OR "Risk Prediction"[Title/Abstract] OR "Risk Screening"[Title/Abstract] OR "Salt Consumption"[Title/Abstract] OR "Secondary Cardiovascular Prevention"[Title/Abstract] OR "Sedentary Behavior"[Title/Abstract] OR "Senolytics"[Title/Abstract] OR "Sleep Apnea"[Title/Abstract] OR "Sleep Disorders"[Title/Abstract] OR "Sleep Health"[Title/Abstract] OR "Smoking"[Title/Abstract] OR "Smoking Cessation"[Title/Abstract] OR "Social Determinants Of Health"[Title/Abstract] OR "Stress Levels"[Title/Abstract] OR "Stress Overload"[Title/Abstract] OR "Stroke Prevention"[Title/Abstract] OR "Training"[Title/Abstract] OR "Wearable-based Phenotyping"[Title/Abstract] OR "Weight Management"[Title/Abstract] OR "Wellness"[Title/Abstract])

BLOCK D– CARDIAC REHABILITATION

("Cardiac Rehabilitation"[Title/Abstract] OR "6-minute Walk Test"[Title/Abstract] OR "Accelerometer"[Title/Abstract] OR "Adherence Rates"[Title/Abstract] OR "Ambulatory Ecg Sensor"[Title/Abstract] OR "Anxiety"[Title/Abstract] OR "Blood Pressure"[Title/Abstract] OR "Body Composition"[Title/Abstract] OR "Body Mass Index"[Title/Abstract] OR "Cardiac Rehabilitation Program"[Title/Abstract] OR "Cardiac Rehabilitation Screening"[Title/Abstract] OR "Cardiac Surgery Rehabilitation System"[Title/Abstract] OR "Cardiological Rehabilitation"[Title/Abstract] OR "Cardiopulmonary Exercise Testing"[Title/Abstract] OR "Cardiovascular Disease"[Title/Abstract] OR "Cardiovascular Patients"[Title/Abstract] OR "Cardiovascular Risk Factors"[Title/Abstract] OR "Chronotropic Incompetence"[Title/Abstract] OR "Clinical Decision Support System"[Title/Abstract] OR "Cloud-based Rehabilitation Dashboard"[Title/Abstract] OR "Coaching"[Title/Abstract] OR "Congenital Heart

Disease"[Title/Abstract] OR "Coronary Artery Disease"[Title/Abstract] OR "Coronary Heart Disease"[Title/Abstract] OR "Depression"[Title/Abstract] OR "Diabetes"[Title/Abstract] OR "Digital Therapeutics Platform"[Title/Abstract] OR "Ejection Fraction"[Title/Abstract] OR "Exercise Prescription"[Title/Abstract] OR "Functional Capacity"[Title/Abstract] OR "Guided Cardiac Rehabilitation"[Title/Abstract] OR "Heart Attack"[Title/Abstract] OR "Heart Failure"[Title/Abstract] OR "Heart Rate Control"[Title/Abstract] OR "Heart Rate Recovery"[Title/Abstract] OR "Heart Rate Variability"[Title/Abstract] OR "Home-based Cardiac Rehabilitation"[Title/Abstract] OR "Hypertension"[Title/Abstract] OR "Ischemic Heart Disease"[Title/Abstract] OR "Lifestyle Modification"[Title/Abstract] OR "Medication Adherence"[Title/Abstract] OR "Metabolic Equivalents"[Title/Abstract] OR "Metabolic Syndrome"[Title/Abstract] OR "Mobile Phone"[Title/Abstract] OR "Motivational Interviewing"[Title/Abstract] OR "Myocardial Infarction"[Title/Abstract] OR "Nutritional Counseling"[Title/Abstract] OR "Optical Flow"[Title/Abstract] OR "Patient Engagement"[Title/Abstract] OR "Patient Fatigue"[Title/Abstract] OR "Patient Portal"[Title/Abstract] OR "Peak Vo_2"[Title/Abstract] OR "Pedometer"[Title/Abstract] OR "Peripheral Vascular Disease"[Title/Abstract] OR "Phase II"[Title/Abstract] OR "Phase III"[Title/Abstract] OR "Physical Activity Counseling"[Title/Abstract] OR "Physiological Parameters"[Title/Abstract] OR "Portable Blood Pressure Monitor"[Title/Abstract] OR "Preventive Medicine"[Title/Abstract] OR "Preserved Ejection Fraction"[Title/Abstract] OR "Psychological Intervention"[Title/Abstract] OR "Psychosocial Assistance"[Title/Abstract] OR "Rehabilitation Outcome"[Title/Abstract] OR "Reduced Ejection Fraction"[Title/Abstract] OR "Risk Factor Management"[Title/Abstract] OR "Robot-assisted Therapy"[Title/Abstract] OR "Secondary Prevention"[Title/Abstract] OR "Smart Bike"[Title/Abstract] OR "Smartphone"[Title/Abstract] OR "Social Functioning"[Title/Abstract] OR "Spirometer"[Title/Abstract] OR "Tele-consultation"[Title/Abstract] OR "Telehealth Platform"[Title/Abstract] OR "Telemonitoring"[Title/Abstract] OR "Telerehabilitation"[Title/Abstract] OR "Ventilatory Threshold"[Title/Abstract] OR "Weight Management"[Title/Abstract])

FULL COMBINED BOOLEAN QUERY

("Artificial Intelligence"[Title/Abstract] OR "Active Learning"[Title/Abstract] OR "Adversarial Robustness"[Title/Abstract] OR "AI Automation"[Title/Abstract] OR "AI Behavioral Science"[Title/Abstract] OR "AI Data Capture"[Title/Abstract] OR "AI Data Privacy"[Title/Abstract] OR "AI Ehealth"[Title/Abstract] OR "AI Ethics"[Title/Abstract] OR "AI Explainability"[Title/Abstract] OR "AI Patient Adherence"[Title/Abstract] OR "AI Patient Monitoring"[Title/Abstract] OR "AI Real-Time Monitoring"[Title/Abstract] OR "AI Regulation"[Title/Abstract] OR "AI Robotics"[Title/Abstract] OR "AI Telemedicine"[Title/Abstract] OR "AI Text Mining"[Title/Abstract] OR "AI-driven Data Analytics"[Title/Abstract] OR "AI-enabled Medical Devices"[Title/Abstract] OR "AI-enabled Smart Phones"[Title/Abstract] OR "Algorithmic Bias"[Title/Abstract] OR "Algorithmic Transparency"[Title/Abstract] OR "Cardiac Imaging Methods"[Title/Abstract] OR "Cardiovascular Imaging"[Title/Abstract] OR "Causal AI"[Title/Abstract] OR "Chatbot"[Title/Abstract] OR "Chatgpt"[Title/Abstract] OR "Computer Vision"[Title/Abstract] OR "Convolutional Neural Networks"[Title/Abstract] OR "Deep Learning"[Title/Abstract] OR "Domain Adaptation"[Title/Abstract] OR "Domain Generalization"[Title/Abstract] OR "Explainable Artificial Intelligence"[Title/Abstract] OR "Federated Learning"[Title/Abstract] OR "Few-shot Learning"[Title/Abstract] OR "Fuzzy Logic"[Title/Abstract] OR "Generative Adversarial Networks"[Title/Abstract] OR "Generative Artificial Intelligence"[Title/Abstract] OR "Graph Neural Networks"[Title/Abstract] OR "Image Analysis"[Title/Abstract] OR "Image Interpretation"[Title/Abstract] OR "K-means Algorithm"[Title/Abstract] OR "Large Language Models"[Title/Abstract] OR "Machine Learning"[Title/Abstract] OR "Medical Imaging"[Title/Abstract] OR "Meta-learning"[Title/Abstract] OR "Mlops"[Title/Abstract] OR "Natural Language Processing"[Title/Abstract] OR "Pattern Recognition"[Title/Abstract] OR "Personalized Medicine"[Title/Abstract] OR "Precision Medicine"[Title/Abstract] OR "Predictive Models"[Title/Abstract] OR "Reinforcement Learning"[Title/Abstract] OR "Self-supervised Learning"[Title/Abstract] OR "Support Vector Machines"[Title/Abstract] OR "Synthetic Data Generation"[Title/Abstract] OR "Transfer Learning"[Title/Abstract] OR "Zero-shot Learning"[Title/Abstract]) AND ("Wearables"[Title/Abstract] OR "Bioelectrical Impedance Sensors"[Title/Abstract] OR "Biosensors"[Title/Abstract] OR "Bluetooth Low Energy Wearable Platforms"[Title/Abstract] OR "Edge Computing In Wearables"[Title/Abstract] OR "Electronic Skin"[Title/Abstract] OR "Energy Harvesting Wearables"[Title/Abstract] OR "Exercise Monitoring Wearables"[Title/Abstract] OR "Flexible/stretchable Electronics"[Title/Abstract] OR "Implantable

Wearables"[Title/Abstract] OR "Internet Of Medical Things"[Title/Abstract] OR "Microneedle Patches"[Title/Abstract] OR "Nfc-enabled Wearables"[Title/Abstract] OR "Photoplethysmography Sensors"[Title/Abstract] OR "Skin-like Electronics"[Title/Abstract] OR "Smart Therapeutic Patches/textiles"[Title/Abstract] OR "Telemonitoring"[Title/Abstract] OR "Vital Sign Monitoring"[Title/Abstract] OR "Wearable Bionsensors"[Title/Abstract] OR "Wearable Devices"[Title/Abstract] OR "Wearable Diagnostics"[Title/Abstract] OR "Wearable Medical Devices"[Title/Abstract] OR "Wearable Robots"[Title/Abstract] OR "Wearable Sensors"[Title/Abstract] OR "Wearable Technology"[Title/Abstract] OR "Wearable Textiles"[Title/Abstract] OR "Wearable Ultrasound Devices"[Title/Abstract] OR "Wireless Body Area Networks"[Title/Abstract]) AND (("Cardiovascular Prevention"[Title/Abstract] OR "Aerobic Exercise"[Title/Abstract] OR "Ai In Risk Stratification"[Title/Abstract] OR "Air Pollution"[Title/Abstract] OR "Alcohol Consumption"[Title/Abstract] OR "Anti-inflammatory Therapy"[Title/Abstract] OR "Arrythmia"[Title/Abstract] OR "Arterial Hypertension"[Title/Abstract] OR "Arterial Motion Monitoring"[Title/Abstract] OR "Artery Calcium Score"[Title/Abstract] OR "Ascvd Risk Score"[Title/Abstract] OR "Atherogenesis"[Title/Abstract] OR "Atherosclerotic Plaque"[Title/Abstract] OR "Behavioral Change Techniques"[Title/Abstract] OR "Blood Glucose Monitoring"[Title/Abstract] OR "Blood Lipid Monitoring"[Title/Abstract] OR "Cancer Treatments"[Title/Abstract] OR "Cardiac Function Tests"[Title/Abstract] OR "Cardiometabolic Diseases"[Title/Abstract] OR "Cardiovascular Digital Twin"[Title/Abstract] OR "Cardiovascular Disease Detection"[Title/Abstract] OR "Cardiovascular Disease Diagnosis"[Title/Abstract] OR "Cardiovascular Disease Phenotyping"[Title/Abstract] OR "Cardiovascular Disease Prediction"[Title/Abstract] OR "Cardiovascular Health Literacy"[Title/Abstract] OR "Cardiovascular Mortality"[Title/Abstract] OR "Cardiovascular Predictive System"[Title/Abstract] OR "Carotid Femoral Pulse"[Title/Abstract] OR "Climate Change"[Title/Abstract] OR "Coronary Disease"[Title/Abstract] OR "Coronary Syndrome"[Title/Abstract] OR "Dietary Patterns"[Title/Abstract] OR "Digital Biomarkers"[Title/Abstract] OR "Dyslipidemia"[Title/Abstract] OR "Early Prevention"[Title/Abstract] OR "Early Risk Factor Detection"[Title/Abstract] OR "Ecg"[Title/Abstract] OR "Environmental Pollution"[Title/Abstract] OR "Exercise Training"[Title/Abstract] OR "Exercising"[Title/Abstract] OR "Family History Of Cardiovascular Disease"[Title/Abstract] OR "Framingham Risk Score"[Title/Abstract] OR "Geoscience"[Title/Abstract] OR "Hba1c"[Title/Abstract] OR "Health Lifestyle"[Title/Abstract] OR "Heart Rate Zones"[Title/Abstract] OR "Heart Stroke"[Title/Abstract] OR "Imaging Biomarkers"[Title/Abstract] OR "Imaging Techniques"[Title/Abstract] OR "Inflammatory Disease"[Title/Abstract] OR "Insomnia"[Title/Abstract] OR "Ldl-cholesterol"[Title/Abstract] OR "Lifestyle Changes"[Title/Abstract] OR "Low-density Lipoprotein"[Title/Abstract] OR "Mediterranean Diet"[Title/Abstract] OR "Mindfullness-based Interventions"[Title/Abstract] OR "Mortality"[Title/Abstract] OR "Motivational Technologies"[Title/Abstract] OR "Multimorbidity"[Title/Abstract] OR "Non-invasive Measurement Techniques"[Title/Abstract] OR "Nutritional Epidemiology"[Title/Abstract] OR "Obesity"[Title/Abstract] OR "Ongoing Monitoring"[Title/Abstract] OR "Oxygen Saturation"[Title/Abstract] OR "Patient Engagement"[Title/Abstract] OR "Peripheral Vascular Disease"[Title/Abstract] OR "Personalized Training Plan"[Title/Abstract] OR "Physical Activity"[Title/Abstract] OR "Physical Inactivity"[Title/Abstract] OR "Plaque Stenosis"[Title/Abstract] OR "Prediction Models"[Title/Abstract] OR "Predictive System"[Title/Abstract] OR "Primary Cardiovascular Prevention"[Title/Abstract] OR "Primary Prevention"[Title/Abstract] OR "Psychosocial Factors"[Title/Abstract] OR "Real-world Data"[Title/Abstract] OR "Remote Patient Monitoring"[Title/Abstract] OR "Resistance"[Title/Abstract] OR "Respiratory Disorders"[Title/Abstract] OR "Risk Evaluation"[Title/Abstract] OR "Risk Factors"[Title/Abstract] OR "Risk Prediction"[Title/Abstract] OR "Risk Screening"[Title/Abstract] OR "Salt Consumption"[Title/Abstract] OR "Secondary Cardiovascular Prevention"[Title/Abstract] OR "Sedentary Behavior"[Title/Abstract] OR "Senolytics"[Title/Abstract] OR "Sleep Apnea"[Title/Abstract] OR "Sleep Disorders"[Title/Abstract] OR "Sleep Health"[Title/Abstract] OR "Smoking"[Title/Abstract] OR "Smoking Cessation"[Title/Abstract] OR "Social Determinants Of Health"[Title/Abstract] OR "Stress Levels"[Title/Abstract] OR "Stress Overload"[Title/Abstract] OR "Stroke Prevention"[Title/Abstract] OR "Training"[Title/Abstract] OR "Wearable-based Phenotyping"[Title/Abstract] OR "Weight Management"[Title/Abstract] OR "Wellness"[Title/Abstract]) OR ("Cardiac Rehabilitation"[Title/Abstract] OR "6-minute Walk Test"[Title/Abstract] OR "Accelerometer"[Title/Abstract] OR "Adherence Rates"[Title/Abstract] OR "Ambulatory Ecg Sensor"[Title/Abstract] OR "Anxiety"[Title/Abstract] OR "Blood Pressure"[Title/Abstract] OR "Body Composition"[Title/Abstract] OR "Body Mass Index"[Title/Abstract] OR "Cardiac Rehabilitation Program"[Title/Abstract] OR "Cardiac Rehabilitation Screening"[Title/Abstract] OR "Cardiac Surgery

Rehabilitation System"[Title/Abstract] OR "Cardiological Rehabilitation"[Title/Abstract] OR "Cardiopulmonary Exercise Testing"[Title/Abstract] OR "Cardiovascular Disease"[Title/Abstract] OR "Cardiovascular Patients"[Title/Abstract] OR "Cardiovascular Risk Factors"[Title/Abstract] OR "Chronotropic Incompetence"[Title/Abstract] OR "Clinical Decision Support System"[Title/Abstract] OR "Cloud-based Rehabilitation Dashboard"[Title/Abstract] OR "Coaching"[Title/Abstract] OR "Congenital Heart Disease"[Title/Abstract] OR "Coronary Artery Disease"[Title/Abstract] OR "Coronary Heart Disease"[Title/Abstract] OR "Depression"[Title/Abstract] OR "Diabetes"[Title/Abstract] OR "Digital Therapeutics Platform"[Title/Abstract] OR "Ejection Fraction"[Title/Abstract] OR "Exercise Prescription"[Title/Abstract] OR "Functional Capacity"[Title/Abstract] OR "Guided Cardiac Rehabilitation"[Title/Abstract] OR "Heart Attack"[Title/Abstract] OR "Heart Failure"[Title/Abstract] OR "Heart Rate Control"[Title/Abstract] OR "Heart Rate Recovery"[Title/Abstract] OR "Heart Rate Variability"[Title/Abstract] OR "Home-based Cardiac Rehabilitation"[Title/Abstract] OR "Hypertension"[Title/Abstract] OR "Ischemic Heart Disease"[Title/Abstract] OR "Lifestyle Modification"[Title/Abstract] OR "Medication Adherence"[Title/Abstract] OR "Metabolic Equivalents"[Title/Abstract] OR "Metabolic Syndrome"[Title/Abstract] OR "Mobile Phone"[Title/Abstract] OR "Motivational Interviewing"[Title/Abstract] OR "Myocardial Infarction"[Title/Abstract] OR "Nutritional Counseling"[Title/Abstract] OR "Optical Flow"[Title/Abstract] OR "Patient Engagement"[Title/Abstract] OR "Patient Fatigue"[Title/Abstract] OR "Patient Portal"[Title/Abstract] OR "Peak Vo_2"[Title/Abstract] OR "Pedometer"[Title/Abstract] OR "Peripheral Vascular Disease"[Title/Abstract] OR "Phase II"[Title/Abstract] OR "Phase III"[Title/Abstract] OR "Physical Activity Counseling"[Title/Abstract] OR "Physiological Parameters"[Title/Abstract] OR "Portable Blood Pressure Monitor"[Title/Abstract] OR "Preventive Medicine"[Title/Abstract] OR "Preserved Ejection Fraction"[Title/Abstract] OR "Psychological Intervention"[Title/Abstract] OR "Psychosocial Assistance"[Title/Abstract] OR "Rehabilitation Outcome"[Title/Abstract] OR "Reduced Ejection Fraction"[Title/Abstract] OR "Risk Factor Management"[Title/Abstract] OR "Robot-assisted Therapy"[Title/Abstract] OR "Secondary Prevention"[Title/Abstract] OR "Smart Bike"[Title/Abstract] OR "Smartphone"[Title/Abstract] OR "Social Functioning"[Title/Abstract] OR "Spirometer"[Title/Abstract] OR "Tele-consultation"[Title/Abstract] OR "Telehealth Platform"[Title/Abstract] OR "Telemonitoring"[Title/Abstract] OR "Telerehabilitation"[Title/Abstract] OR "Ventilatory Threshold"[Title/Abstract] OR "Weight Management"[Title/Abstract]))

OPERATIONAL DETAILS

Database: PubMed (MEDLINE).

Fields: Title/Abstract

Filters: The PubMed strategy was limited using predefined filters to increase relevance and feasibility. Specifically, results were restricted to the last five years, to articles available in full text, and to studies involving human subjects. In addition, only publications in English and Spanish were considered. These restrictions were applied to balance comprehensiveness with feasibility of screening, while ensuring inclusion of the most recent and clinically relevant literature.

Preliminary search date: 25/09/2025

Preliminary Results: Preliminary searches retrieved 1255 records in PubMed (September 2025); final counts will be reported in the PRISMA flow diagram of the scoping review.

Adaptation: The same block structure will be adjusted for Web of Science, IEEE Xplore, and CINAHL.
